# Nucleocapsid Structure of Negative Strand RNA Virus

**DOI:** 10.3390/v12080835

**Published:** 2020-07-30

**Authors:** Ming Luo, James Ross Terrell, Shelby Ashlyn Mcmanus

**Affiliations:** Department of Chemistry, Georgia State University, Atlanta, GA 30302, USA; jterrell18@student.gsu.edu (J.R.T.); smcmanus3@student.gsu.edu (S.A.M.)

**Keywords:** negative strand RNA virus, nucleocapsid, viral RNA-dependent RNA polymerase, cofactor, cross subunit interactions, capsid protein motif, 5H+3H

## Abstract

Negative strand RNA viruses (NSVs) include many important human pathogens, such as influenza virus, Ebola virus, and rabies virus. One of the unique characteristics that NSVs share is the assembly of the nucleocapsid and its role in viral RNA synthesis. In NSVs, the single strand RNA genome is encapsidated in the linear nucleocapsid throughout the viral replication cycle. Subunits of the nucleocapsid protein are parallelly aligned along the RNA genome that is sandwiched between two domains composed of conserved helix motifs. The viral RNA-dependent-RNA polymerase (vRdRp) must recognize the protein–RNA complex of the nucleocapsid and unveil the protected genomic RNA in order to initiate viral RNA synthesis. In addition, vRdRp must continuously translocate along the protein–RNA complex during elongation in viral RNA synthesis. This unique mechanism of viral RNA synthesis suggests that the nucleocapsid may play a regulatory role during NSV replication.

## 1. Introduction

All viruses assemble a nucleocapsid. The capsid consists of viral proteins and encloses the nucleotide genome of the virus. The word “capsid” is originated from the Latin *capsa* (box). The primary function of the capsid (also known as “protein coat” or “protein shell”) is to carry and protect the viral genome during transmission between cells. The term “nucleocapsid” refers to the capsid protein–nucleotide complex. For efficiency, the viral nucleocapsid is assembled by organization of capsid protein subunits following a geometric symmetry, including the icosahedral symmetry for spherical viruses and the helical symmetry for filamentous viruses (read Chapter 3 in Fields Virology for details) [1]. For negative strand RNA viruses (NSVs), the nucleocapsid has a unique structure that is pertinent to its functions in the virus replication cycle. This review will discuss the assembly of the NSV nucleocapsids and the structure–function relationship.

According to International Committee on Taxonomy of Viruses (ICTV), NSVs belong to Phylum *Negarnaviricota*, Realm: *Riboviria* [2,3]. Since 2003, the structure of the nucleocapsid or the capsid protein has been determined for at least 21 genera in *Negarnaviricota* (Table 1). The determined structures have confirmed a genetic relationship among members of different NSV families. Common folds of the NSV capsid proteins and principles of nucleocapsid assembly have emerged, and the functional role of the nucleocapsid in the unique NSV viral RNA synthesis has also become clearer. It is because of this role that additional examination of the structure of NSV nucleocapsids has now become essential for further understanding of NSV replication and pathogenesis.

## 2. The Capsid Protein Fold

The Phylum *Negarnaviricota* has been divided into two Subphyla; *Haploviricotina* and *Polyploviricotina.* The distinction between the two Subphyla is the nonsegmented or segmented nature of the viral genome. The NSVs are then further classified phylogenetically ultimately yielding a separation by virion morphology. Although these viruses can differ greatly in their global structures, structural and functional similarities are observed when comparing their capsid proteins.

**Figure 1:**
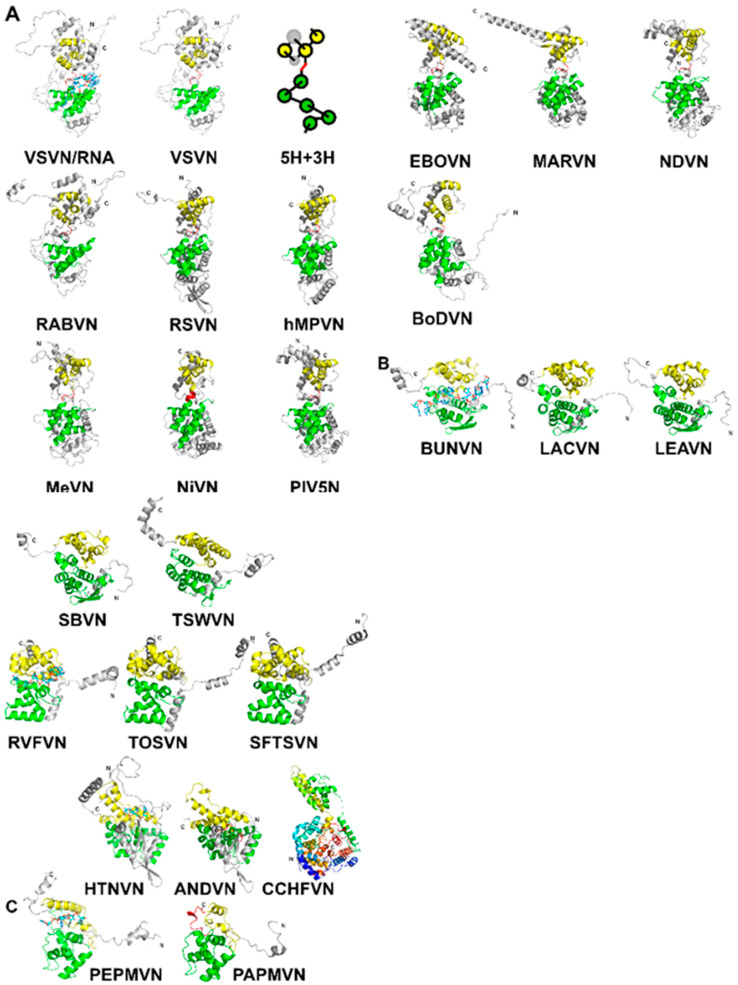
(**A**) A ribbon drawing of VSV nucleocapsid protein (VSVN) in complex with 9 nucleotides (sticks) encapsidated. The 5H in the N-lobe is colored green, and 3H in the C-lobe is colored yellow. Termini are labeled as N and C, respectively. The linker between the two lobes is colored red. The same ribbon drawing without RNA is shown to the right. Ribbons drawings in this and the following figures are prepared with PyMol [40]. A cartoon is drawn to illustrate the 5H+3H motif. Ten other related N structures in Table 2 are also shown using the same color scheme. (**B**) Eleven N structures in *Polyploviricotina* are shown, with those from BUNV (BUNVN), RVFV (RVFVN), and HTNV (HTNVN) in complex with encapsidated nucleotides (sticks). The N-domain in the N core is colored green and the C-domain in the N core is colored yellow. Structures of closely relayed N proteins according to Table 3 and Table 5 are grouped together. The structure from CCHFV (CCHFVN) is colored rainbow from the N-terminus (blue) to the C-terminus (red) because no clear N- and C-domains could be identified. (**C**) Two structures from the +ss RNA viruses are shown, with that from PepMV (PepMVN) in complex with encapsidated RNA. The color scheme is the same as in (**B**).

The capsid proteins (N) of NSVs share a common fold. As shown for vesicular stomatitis virus (VSV) in Figure 1A, the N-terminal lobe and the C-terminal lobe are connected by a single polypeptide chain [14]. The core of the capsid protein consists of five α-helices in the N-terminal lobe (5-H motif) and three α-helices in the C-terminal lobe (3-H motif) [34]. When the protein subunit is assembled into the nucleocapsid, the encapsidated RNA is situated between the two lobes. The observation of a conserved structural motif present in all nucleocapsid proteins in NSVs is analogous to that a β-barrel fold is observed in the capsid proteins of spherical viruses [35].

The two largest NSV orders contain the majority of known capsid protein structures are *Mononegavirales* and *Bunyavirales*. Beyond these, there are a small number of structures from *Articulavirales* represented by the segmented *Orthomyxoviruses*. Structures from the order *Alphaflexiviridae* are included in this review because of structural similarities, even though these are positive single strand RNA viruses.

The structures of *Haploviricotina* capsid proteins from 11 members in the order *Mononegavirales* were superimposed using the program Fr-TM-align [36] (Table 2). Large variance in capsid protein Size is observed in known structures with MWs ranging from 41 kDa in BoDV up to 83.3 kDa in EBOV; however, the capsid cores maintain structural homology. Similarities among these structures are essentially the same as previously observed [34,37]. It is well expected that viruses in the same family have a high structural similarity in their capsid proteins, such as VSV and RABV; RSV and hMPV; PIV5, MeV, NiV, and NDV; EBOV and MARV (Figure 1A). However, the observed similarity between NDV and MARV appears to be an exception (Table 2). The nucleocapsid structure of MARV is very close to that of EBOV. The apparent more favorable alignment for MARV could be due to that less residues are included in the structure of MARV nucleocapsid protein. 

For *Polyploviricotina* NSVs, homology is only present among members in the same family (Table 3 and Table 4). However, the fold of the capsid proteins shares some common features with those from members in *Mononegavirales*. The most apparent similarity is that the encapsidated genomic RNA is also sandwiched between the N- and C-terminal lobes (Figure 1B).

Further examination reveals that the 5-H and 3-H motifs share the same topology, but the orientations of the helices are often different. For members in *Peribunyaviridae* and *Tospoviridae*, the N-terminal lobe contains the same 5-helices and may be superimposed with that from members in *Mononegavirales* (Table 5). For the C-terminal lobe, on the other hand, the first two helices in the 3-H motif could be superimposed with those from members in *Mononegavirales* while the third α-helix takes a reverse orientation. For *Bunvaviridae* members in *Phenuiviridae, Nairoviridae,* and *Hantaviridae*, the helices in the N-terminal 5-H motif have large differences in their orientation despite the same topology. Their N-terminal lobes could not be superimposed with those from members in *Mononegavirales*. The same situation is found in the C-terminal 3-H motif. However, the fold in the C-terminal lobe from members in *Nairoviridae* and *Hantaviridae* is homologous to that from members in *Peribunyavirida*, except for the orientation of the last α-helix (Table 5).

Finally, the size of the RNA cleft in a N subunit may vary widely in these viruses. The C-terminal lobe from members in *Bunyavirales* has much less separation from the N-terminal lobe so that the cleft between the two lobes is much narrower than that from members in *Mononegavirales*. This narrowing of the RNA cleft results in a very shallow pocket in which the RNA resides in a fissure rather than within a deep cavitation [21]. Differences in these structural features may allow more a flexibility in supramolecular nucleocapsid morphology to package the segmented genomes that are incorporated stochastically during virion assembly [38,39].

For members in *Orthomyxoviridae*, direct structural comparison of the nucleocapsid proteins with other families is not informative; two terminal lobes are visible, but the division is not completely clear because no information of RNA encapsidation is available [28].

In addition to NSVs, the structure of nucleocapsid proteins from members in *Alphaflexiviridae* is included in the comparison even though they are positive single strand RNA viruses [32,33]. They contain similar N- and C-terminal lobes that encapsidate the genomic nucleotide between them (Figure 1C). Some of the bases in the RNA strand are stacked and face the exterior of the nucleocapsid, whereas some face the interior. The N-terminal domain of their N proteins is homologous to that of NSV N proteins (Table 5). This suggests that the strategy to assemble a linear nucleocapsid is common in both negative and positive single strand RNA viruses, and the same structural features in the capsid protein are essential for the encapsidation of the linear nucleotide in the nucleocapsid.

## 3. Assembly of the Nucleocapsid

The structure of VSV nucleocapsid was first solved as a nucleocapsid-like particle (NLP) [14]. The structure of other NLPs and nucleocapsids solved subsequently shares the same scheme of nucleocapsid assembly (Table 1).

**Figure 2:**
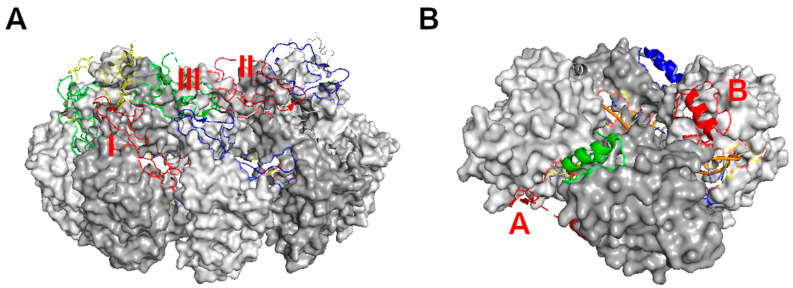
Linear assembly of NSV nucleocapsids. (**A**) Assembly of VSV nucleocapsid represented by five subunits (colored respectively) [14]. The encapsidated RNA (45 nucleotides) is represented by an orange ribbon and blue sticks. The cross subunit interactions between the red N-terminus and the green C_lobe (named contact I); between the red C_loop and the blue C_lobe (named contact II); and between the green C_loop and the blue N-terminus (named contact III) are labeled with orange letters. (**B**) Assembly of LACV nucleocapsid represented by four subunits (colored respectively) [21]. The encapsidated RNA (44 nucleotides) is represented by an orange ribbon and blue sticks. The cross subunit interactions between the red N-terminus and the green subunit (named contact A); and between the red C-terminus and the blue subunit (named contact B) are labeled with red letters.

First, the subunits of the nucleocapsid protein are parallelly aligned along the linear RNA genome. Unlike the icosahedral symmetry in the spherical viral nucleocapsid, this one dimensional linear symmetry allows the genomic RNA to be encapsidated at any length and to be fully protected (Figure 2). Although some NSV nucleocapsids appear to be helically coiled, this is different from a helical nucleocapsid, such as that of tobacco mosaic virus, that assembles following a strict helical symmetry. The helical coil of NSV nucleocapsids is not required for RNA encapsidation and the apparent helical symmetry changes with the environmental conditions and from virion to virion [41,42].

Second, the encapsidated genomic RNA is accommodated in the cleft formed between the N- and C-terminal lobes. The genomic RNA assumes a fixed three dimensional structure in the protein subunit with bases stacked similarly as one strand in an A-form RNA duplex. However, it should be noted that the bases are stacked as separated fragments with bases in one fragment facing the interior of the cleft and bases in the next fragment facing the opening of the cleft. The nucleotides situated between subunits may possess a more flexible conformation. Some of the backbone phosphate groups in the genomic RNA may be coordinated with positively charged residues in the cleft, but there are no defined motifs as found in specific RNA binding proteins [43].

Third, the assembly of the nucleocapsid is stabilized mainly by interactions between the subunits of nucleocapsid protein. This is proved by the fact that a similar capsid could be assembled in the absence of RNA [44]. There are extensive contacts between neighboring subunits with broader contacts between the C-lobes than the N- [34]. Without these contacts, the subunits of the nucleocapsid protein were unable to assemble as shown by mutational studies [44]. The requirement for encapsidation of the genomic RNA in the nucleocapsid is to form a stable capsid, which is consistent with the observation that the nucleocapsid protein does not possess an RNA binding motif. In addition to the contacts between the subunits, there are extensive cross subunits interactions through the extended termini or large loops in the subunit. In VSV, the N-terminus of one subunit interacts with the C-lobe of +1 subunit in the 5′ direction, and the large loop in the C-lobe of +2 subunit, whereas its own large loop in the C-lobe interacts with the C-lobe in −1 subunit. All interactions between the subunits are required for the assembly of the nucleocapsid [44].

This assembly scheme of linear nucleocapsid is maintained in all members in the order *Mononegavirales* with limited variations. In RSV and hMPV, there are seven nucleotides associated with each subunit rather than the nine seen in VSV. In PIV5, MeV, NDV, EBOV, and MARV, there are six nucleotides associated with each subunit. The nucleocapsid structure of NiV and BoDV was determined in the absence of RNA. A notable difference in these viruses is that the C-terminus instead of a large loop in the C-lobe is involved in cross subunit interactions.

In *Orthobunyaviruses*, there are 11 nucleotides associated with each subunit (Figure 2B). The 11th> nucleotide is situated between subunits and seems to be stabilized only by RNA interactions. In *Phleboviruses*, there are also seven nucleotides associated with each subunit, with two nucleotides stabilized by RNA interactions at the subunit boundary. The cross subunit interactions are mainly through the N-terminus with almost no contribution from the C-terminus. In Hantaan virus, there are three nucleotides per subunit. The cross subunit interactions are much more extensive than nucleocapsids from other viruses [17]. Each subunit has domain interchanges with +3 and -3 subunits, including residues in termini and five protruding loops.

For members in *Bornaviridae* and *Orthomyxoviridae*, the nucleoprotein structure was solved without encapsidated RNA. Cross subunit interactions were observed, but how they are related to the interactions and RNA encapsidation in the nucleocapsid remains to be elucidated. The nucleocapsid structure of influenza A virus was solved by cryoEM image reconstruction [45,46]. Its linear nucleocapsid possesses the same general characteristics as other NSVs, but with the double-twist. The 3′ and 5′ termini of the RNA genome are associated with the viral RNA-dependent RNA polymerase so that each genome segment of the double-twist nucleocapsid is associated with one polymerase complex to form the ribonucleoprotein complex (RNP) [47]. The structure of the nucleocapsid protein from *Mammarenavirus* (LASV) or *Orthotospovirus* (TSWV) was solved as a protein–RNA complex. However, these structures are not NLPs and the protein–RNA interactions observed in these structures reflect portions of those interactions in the nucleocapsid. For members in *Nairovirida*, the structures were protein monomers.

**Figure 3:**
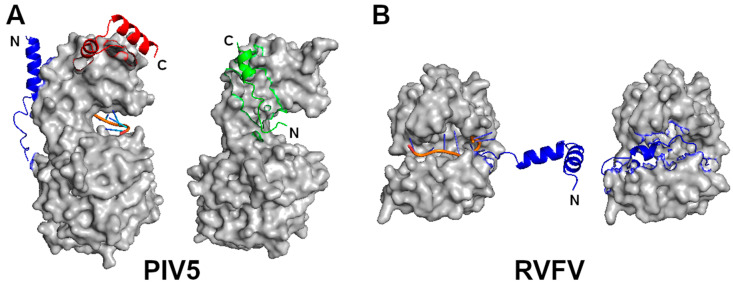
Monomeric N proteins. (**A**) A N subunit in the NLP of PIV5 (left) is compared with that of N–P complex (right). The two structures are superimposed by use of the C-lobes. The RNA is represented by an orange ribbon and blue sticks. The blue and red regions represent the N- and C-termini, respectively as noted by N and C, in the N-RNA complex. The green polypeptide in the N–P complex corresponds to residues 7–25 in the P protein. Its N- and C-termini are noted by N and C, respectively. The P polypeptide occupies the site where the blue N-terminus of the neighboring N subunit is located in the nucleocapsid, even though it has an opposite orientation. (**B**) A N subunit in the NLP of RVFV (left) is compared with that of RNA free N (right). The blue region represent the N-terminal residues 1–34. The RNA is shown in the same color scheme.

The assembly of the nucleocapsid takes place while the new genomic RNA is replicated for both the viral RNA (- sense) and complimentary RNA (+ sense) genomes. Before the nucleocapsid subunit is incorporated in the nucleocapsid, it has to remain as a monomer and RNA free. Several mechanisms may be employed by different NSVs. For members in *Paramyxoviridae, Pneumoviridae,* and *Rhabdoviridae,* there is a virus-coded chaperone protein, called phosphoprotein (P) because of its heavy phosphorylation, that binds the N protein to keep it monomeric and RNA free (Figure 3A). For members in *Rhabdoviridae*, there are two P binding sites in the N protein. First, a region in the N-terminus of the P protein binds in the cleft of the N protein where the RNA is accommodated in the nucleocapsid [48]. Second, the C-terminal domain of the P protein binds the extended loop in the C-terminal lobe of the N protein. Through these two interactions, the P protein can prevent the genomic RNA from residing in the N protein cleft and the polymerization of the N protein by blocking the side-by-side contact found in the nucleocapsid [34]. For members in *Paramyxoviridae* and *Pneumoviridae,* a region in the N-terminus of the P protein binds at a site in the N protein that is involved in cross subunit interactions in the assembled nucleocapsid [8,11,49]. The P N-terminus is extended to the RNA cleft in the N protein. In addition, the P protein may stabilize the cleft in the N protein allowing it to maintain an open conformation. For members in *Filoviridae*, a region in the N-terminus of VP35, a P protein homolog, binds the N protein to prevent its polymerization and the N protein chaperoned by the VP35 protein also maintains the open conformation [50,51]. It is clear that prevention of N protein polymerization is the main mechanism to keep the N protein RNA free before being incorporated into the nucleocapsid during assembly. This is further supported by that the encapsidation of RNA in the nucleocapsid was inhibited by mutating residues in the side-by-side contact of the N proteins with no changes in the cleft of the N protein [44].

In *Peribunyaviruses,* the two termini of the N protein are highly flexible in the monomer^2^. They become well ordered through cross subunit interactions in the nucleocapsid. In *Phenuiviruses*, on the other hand, the N-terminus folds back in the cleft when the N protein is monomeric and becomes extended to interact with the neighboring subunit in the nucleocapsid [24,52] (Figure 3B). In the monomeric N protein of influenza virus, the only structural change is in the extended loop that is involved in the cross subunit interactions in the oligomer [53]. However, it is also possible that the N-terminus folds back in the cleft of the N protein by comparison to a homologous N protein from a related infectious salmon anemia virus [31]. Nevertheless, the N protein from these viruses manages to remain monomeric by assuming highly flexible conformations to avoid polymerization without binding by a viral chaperone protein.

The encapsidation of genomic RNA in the nucleocapsid occurs concomitantly with viral RNA replication. This requires that monomeric N subunits are delivered at the replication site to encapsidate the genomic RNA as it emerges from the polymerase. For *Peribunyaviruses* and *Phenuiviruses*, the N protein can remain as a monomer without a viral chaperone protein. It may defuse to the replication site to encapsidate the genomic RNA emerging from the replication site of the viral polymerase. It is likely that there is a docking site on the polymerase for the N protein although there is no direct experimental data to support this notion. The N protein monomer is more easily added onto the previous N subunit through N-N interactions when the nucleocapsid is elongated. For viruses that have a viral chaperone protein, the assembly process may be facilitated in two ways. First, the binding site for the viral chaperone protein is on the side of the N protein that is not the contact surface when the incoming N subunit is added to the elongating nucleocapsid, i.e., the 3′ side. The viral chaperone protein is then released by conformational changes in the assembled N protein subunit. This mechanism ensures that the monomeric N protein is added to the elongating nucleocapsid only from the correct side. Second, the viral chaperone protein may also be involved in the docking of N at the replication site by its direct interactions with the viral polymerase. For instance, the structure of the VSV phosphoprotein (P) showed that there are direct interactions between dimers of the P protein [54]. It is possible that the chaperone P protein helps to correctly deliver the monomeric N protein by interacting with the polymerase cofactor P protein [55].

## 4. Viral RNA Synthesis

One of the unique features in viral RNA synthesis of NSVs is that the template is the nucleocapsid, not the naked genomic RNA [56]. This requires that the initiation, elongation and termination of viral RNA synthesis must be completed with the viral RNA genome always inside the nucleocapsid during the process. The viral polymerase, the nucleocapsid protein and the genomic RNA must function together to carry out the viral RNA synthesis.

It has been long recognized that the nucleocapsid protein plays an essential role in the viral RNA synthesis of NSVs. In paramyxoviruses such as Sendai virus, there is so called “rule of six” that stipulates that the viral genome is only replicated efficiently when the genome length is 6n nucleotides (n is an integral number) [57]. This indicates that each nucleocapsid protein must cover 6 nucleotides to allow the nucleocapsid to serve as an efficient template. Indeed, the structure of the nucleocapsid or NLP for MeV, PIV5, and NDV confirmed that six nucleotides are covered by each subunit. The promoter for replication is composed of the hexamer sequences associated with N protein subunits [58].

Other features also suggest that the nucleocapsid protein plays a role in viral RNA synthesis. It has been observed that RNA editing occurs at conserved sites during mRNA transcription of certain paramyxoviruses. In Sendai virus, this is 3′ UAA UUUUUU CC*C in which additional Gs may be inserted at C* during mRNA transcription [59]. During this process, the viral polymerase needs to backtrack at this site during transcription, also known as stuttering. A similar process also occurs during polyA tail synthesis during transcription. At the end of each ORF in the genome of NSV, there is a track of Us. In the final step of transcription, the viral polymerase stutters at the U track and synthesizes a polyA tail before termination of transcription. Stuttering during viral transcription is clearly associated with the role of the nucleocapsid protein in viral RNA synthesis. For instance, stuttering is bypassed when the P binding site in the N protein of VSV was mutated to produce a readthrough transcript [60]. The stuttering function was restored when the U track was extended by an additional U in rescued VSV [61]. These results firmly demonstrate that the nucleocapsid protein is an indispensable component in viral RNA synthesis of NSV.

Since the viral genome is encapsidated in the nucleocapsid during viral RNA synthesis, interactions of the RNA sequence with the nucleocapsid protein may be involved in regulation of polymerase activities. One piece of evidence could be related to the unusual codon usage bias by the genome of NSV. For instance, the pattern of relative synonymous codon usage is highly conserved throughout the influenza A virus subtypes, suggesting the integrity of viral RNA structures must be maintained for efficient viral replication [62]. In strains of Ebola virus, highly preferred codons are all A-ending triplets and the most abundant tRNAs present in the human cells are not used preferentially, indicating that factors other than the efficiency of protein translation selected the codon usage bias [63]. This observation is further expanded in 13 *Mononegavirales* species in that the level of gene expression, i.e., viral transcription, is the key determinant of gene compositions [64]. In a mutant VSV in which the codon pair bias was altered in the L protein of the polymerase, its virulence in mice was attenuated without compromising viral protein translation [65]. The rate of changes in codon usage bias was found much lower than the error rate of the paramyxovirus polymerase, confirming that mutation of the genomic RNA sequence is limited by mechanisms other than evolutionary pressure on protein sequences [66].

To define which step in viral RNA synthesis is mostly affected by the genomic RNA sequence, the codon usage preference of VSV genome was mutated to that of the mammalian host [67]. The most profound impact is the reduction of viral transcription levels, consistent with previous observations. The effects on initiation or termination of transcription are more obvious. Analyses by kappa index of coincidence (a number to indicate a degree of association not by random) revealed that clusters of purine or pyrimidine nucleotides are reciprocally related to the level of RNA transcription. By thermal shift assays of nucleocapsid stability, it was shown that a nucleocapsid-like particle (NLP) containing poly(rA) is more stable than that containing random RNA sequences [67]. This is consistent with the observation that a compound stabilizing the nucleocapsid also inhibits viral RNA synthesis by VSV polymerase [68]. On the other hand, an NLP containing poly(rU) is less stable than that containing random RNA sequences, suggesting polymerase stuttering may be related to instability of the nucleocapsid.

Ultimately, the viral synthesis during NSV replication may proceed only when a productive complex is formed between the nucleocapsid and the viral polymerase. The structure of the polymerase in complex with the promoter of influenza virus has been reported [47,69,70,71]. The polymerase of influenza virus is composed of three subunits, PA, PB1, and PB2, and the active site of the viral RNA synthesis is located in PB1. In all genome segments of influenza virus, the 3′ and 5′ ends form a unique structure containing a duplex and a stem–loop motif. A polymerase complex is associated with the promoter in each segment and copackaged in the virion. The structure of the polymerase bound with the promoter clearly marked where the 3′ template is placed and the tunnels for NTP entry and product exit [72,73]. This represents the initiation complex for viral synthesis and its topology is similar to the polymerase from other RNA viruses [74]. A similar structure has also been shown for the viral polymerase of segmented LACV even though the LACV polymerase is a single polypeptide that aligns with the three subunits of influenza virus polymerase [75]. A model for elongation in viral RNA synthesis has been proposed, but it did not address how the polymerase could gain access to the genomic RNA template still encapsidated in the nucleocapsid.

**Figure 4:**
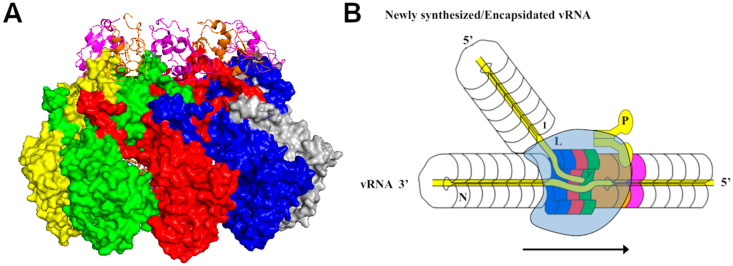
Mechanism of viral RNA synthesis by NSV. (**A**) Recognition of the nucleocapsid template by the cofactor P of VSV viral RNA-dependent-RNA polymerase (vRdRp) [78]. Five N subunits are colored yellow, green, red, blue and gray, respectively. The nucleocapsid binding domain of the P protein is colored magenta or orange. One magenta P subunit sits at the top between the C-lobes of the green and red N subunits. (**B**) A cartoon for a model of the VSV viral RNA replication. The L (light blue) and P subunits (yellow) in VSV vRdRp form an activity bubble by opening N subunits (blue, red and green) to reveal the genomic RNA. The newly synthesized viral RNA exits the L subunit and is encapsidated concomitantly by incoming N subunits. The activity bubble is translocated from the 3′ end to the 5′ end, opening the N subunits (brown and purple) and leaving the N subunits (blue and red) behind to close on the genomic RNA.

For members in *Mononegavirales*, the viral polymerase is composed of the large subunit (L) that contains the active site, and a cofactor. In most families, the cofactor is named the phosphoprotein (P) due to its heavy phosphorylation. In *Filoviridae*, the cofactors are VP35, a homolog of the P protein, and VP30 required for viral transcription [76,77]. The P protein plays a double-role. It functions as a chaperone protein to keep monomeric N protein RNA-free, as discussed above. As a cofactor, the P protein bridges the interaction of the L protein with the nucleocapsid template. In order to fulfill different roles, the interactions between the P and N proteins may be different in different steps in NSV replication. As discussed above, the P protein has a chaperone function to keep the monomeric N protein RNA-free. The P binding sites for the chaperone function are located to where the interactions between the N proteins which are required for the nucleocapsid assembly, or where the encapsidated RNA is located. Blocking these sites will prevent the monomeric N protein from premature assembly with random RNA. On the other hand, the binding site for the cofactor function must be present only in the nucleocapsid in order for the polymerase to recognize the nucleocapsid. In the nucleocapsid of Rhabdovirus VSV, the P binding consists of residues from two neighboring N subunits [78] (Figure 4A). This guarantees that the cofactor binding site will be constructed only when the nucleocapsid is assembled. In paramyxoviruses, the extreme C-terminal region of the P protein interacts with an α-helix in the tail of the N protein, named α-MoRE (molecular recognition element) [79,80,81]. Despite MoRE being a key element in P binding, the more complete binding site on the nucleocapsid is located between two neighboring N subunits [82], similar to that in Rhabdovirus. This indicates that the full binding of the P protein on the nucleocapsid is required for bringing the L protein to the nucleocapsid for viral RNA synthesis. Furthermore, the N-terminal domain of mumps virus, a Rubulavirus, also binds the nucleocapsid and induces relaxation of the helical nucleocapsid to facilitate viral RNA synthesis [82,83].

There are two types of viral RNA synthesis: transcription and replication. In an NSV genome that encodes more than one viral protein, the promotor for transcription is located at the internal gene junction. The structure of the L-P complexes [55,84,85,86] showed that the L protein is stabilized in the conformation prior to initiation by the bound P protein. The nascent RNA may exit to activate the capping and methyltransferase so that the elongation of mRNA will proceed [87]. In the presence of RNA-free N, the nascent RNA can exit the L-P complex, bypassing the capping enzyme, and be encapsidated by incoming N protein subunits that can be delivered as a N–P complex to complete elongation of genome replication [88,89].

For members in *Mononegavirales*, the promoter for viral replication is at the 3′ end and the promoters for viral transcription are internal at the gene junctions. How the viral polymerase may recognize the different promoters is not entirely clear. Unlike in influenza virus, multiple copies of the viral polymerase are packaged in the virion [90], but not bound with the 3′ promoter [42]. It was suggested that the replicase that recognizes the 3′ promoter is composed of L, P and N proteins [88]. However, it is still an open question how the 3′ promoter could be recognized by a replicase. The atomic structure of the VSV L protein has been determined by cryoEM image reconstruction [91]. The active site in the L protein can be clearly identified by homology to other viral RNA polymerase, and the tunnels for the template, NTPs and the product could also be identified. At the same time, the structure of the isolated L protein suggests that a significant conformational change needs to occur in order to allow the RNA template to be placed in the active site. The structural change of the L protein may be induced by interactions with the P protein (the cofactor), a N protein (a component of the replicase), the nucleocapsid (the template), or most likely, a combination of all these interactions.

Since the template RNA is encapsidated in the nucleocapsid, it must be made available to the active site of the polymerase during viral RNA synthesis. Two elements are essential to this process: First, a conformational change must be induced in the N protein subunits to release the encapsidated RNA, and second, the integrity of the nucleocapsid must be restored after RNA synthesis is completed. Several structures showed that the P protein chaperoned N monomer has an open conformation in comparison with that in the nucleocapsid [8,49], but others have a more closed or unchanged conformation [50,51,92]. It is unlikely that a global structural change in the N protein is induced in order to reveal the encapsidated RNA, which may also lead to disruption of the nucleocapsid. In fact, structural changes are observed in the helices that gate the encapsidated RNA when the structure of the nucleocapsid is compared with that of the empty capsid or RNA-free N protein [15,93]. It has been proposed that the active complex for viral RNA synthesis is formed like a bubble by close association of the viral polymerase with the nucleocapsid [78] (Figure 4B). The encapsidated RNA is locally released from the N protein subunits, with local structural changes induced by the polymerase. During elongation, this activity bubble is translocated along the nucleocapsid and the structure of the nucleocapsid is restored after the bubble passes through. This model is consistent with the observation that the stability of the nucleocapsid regulates the activity of viral RNA synthesis [67,94]. Indeed, a compound that stabilizes the nucleocapsid inhibits replication of VSV [68]. Mutations in the gating α-helix of VSV N protein diminished viral RNA synthesis and compensatory mutations to restore the activity were found in the viral polymerase [95].

## 5. Conclusions

The nucleocapsid structure of NSV has many common features as found in spherical viruses [96]. In spherical viruses, the most common fold in the capsid protein is the β-barrel [97]. In the nucleocapsid protein of NSVs, the conserved fold is the V-shaped 5H+3H motif. Analogous to the spherical viruses that follow the icosahedral symmetry to assemble the virion, the nucleocapsid of NSV is assembled by linear alignment of the N protein subunits associated through side-by-side and cross-molecule (also known as “domain swap”) interactions. The linear symmetry of the nucleocapsid matches the requirement of NSV viral RNA synthesis in which the template is the protein–RNA complex of the nucleocapsid. All steps of viral RNA synthesis, including initiation, elongation and termination, must be accomplished while the genomic RNA remains encapsidated in the nucleocapsid. During this process, the viral polymerase needs to unveil the encapsidated RNA template and translocate along the protein–RNA complex of the nucleocapsid. Further studies of these intimate interactions will reveal the unique mechanism of viral RNA synthesis in replication of NSVs.

## Figures and Tables

**Table 1:** Reported nucleocapsid structure of members from Phylum *Negarnaviricota.*

Family	Subfamily	Genus	Name	(PDB Code)
**Subphylum:** ***Haploviricotina*;** **Class:** ***Monjiviricetes*;** **Order:** ***Mononegavirales***				
*Bornaviridae*		*Mammalian 1 orthobornavirus*	Borna disease virus (BoDV)	(1N93) [4]
*Filoviridae*		*Ebolavirus*	Ebola virus (EBOV)	(4Z9P) [5]
		*Marburgvirus*	Marburg virus (MARV)	(5F5M) [6]
*Paramyxoviridae*	*Avulaviridae*	*Avian orthoavulavirus 1*	Newcastle disease virus (NDV)	(6JC3) [7]
	*Orthoparamyxovirdae*	*Henipavirus*	Nipah virus (NiV)	(4CO6) [8]
		*Morbillivirus*	Measles virus (MeV)	(4UFT) [9]
	*Rubulaviridae*	*Orrthorubulavirus*	parainfluenza virus 5 (PIV5)	(4XJN) [10]
*Pneumoviridae*		*Metapneumovirus*	Human metapneumovirus (hMPV)	(5FVC) [11]
		*Orthopneumovirus*	respiratory syncytial virus (RSV)	(2WJ8) [12]
*Rhabdoviridae*		*Lyssavirus*	Rabies virus (RABV)	(2GTT) [13]
		*Vesiculovirus*	Vesicular stomatitis virus (VSV)	(2GIC) [14]
**Subphylum:** ***Polyploviricotina*;** **Class:** ***Ellioviricetes*;** **Order:** ***Bunyavirales***				
*Arenaviridae*		*Mammarenavirus*	Lassa Virus (LASV)	(3T5Q) [15]
*Hantaviridae*	*Mammantaviridae*	*Orthohantavirus*	Andes virus (ANDV)	(5E04) [16]
			Hantaan virus (HTNV)	(6I2N) [17]
*Nairoviridae*		*Orthonairovirus*	Crimean-Congo Hemorrhagic Fever Virus (CCHFV)	(4AKL) [18]
			Hazara virus (HAZV)	(4XZE) [19]
			Kupe virus (KUPV)	(4XZC) [19]
			Erve virus (ERVEV)	(4XZ8) [19]
*Peribunyaviridae*		*Orthobunyavirus*	Bunyamwera virus (BUNV)	(4IJS) [20]
			La Crosse virus (LACV)	(4BHH) [21]
			Leanyer orthobunyavirus (LEAV)	(4J1G) [22]
			Schmallenberg virus (SBV)	(4JNG) [23]
*Phenuiviridae*		*Phlebovirus*	Rift Valley Fever Virus (RVFV)	(4H5P) [24]
			Toscana virus (TOSV)	(4CSF) [25]
			Severe fever with thrombocytopenia syndrome virus (SFTSV)	4J4R) [26]
*Tospoviridae*		*Orthotospovirus*	Tomato spotted wilt tospovirus (TSWV)	(5IP3) [27]
**Subphylum:** ***Polyploviricotina*;** **Class:** ***Insthoviricetes*;** **Order:** ***Articulavirales***				
*Orthomyxoviridae*		*Alphainfluenzavirus*	Influenza A virus (IFAV)	(2IQH) [28]
		*Betainfluenzavirus*	Influenza B virus (IFBV)	(3TJ0) [29]
		*Deltainfluenzavirus*	Influenza D virus (IFDV)	(5N2U) [30]
		*Isavirus*	Infectious Salmon Anemia Virus (ISAV)	(4EWC) [31]
	**Class:** *Tymovirales* *; **Order:** ***Alphaflexiviridae***			
		*Potexvirus*	Papaya mosaic virus (PapMV)	(4DOX) [32]
			Pepino mosaic virus (PepMV)	(5FN1) [33]

* *Tymovirales* include viruses that have (+) sense single stranded RNA genomes.

**Table 2:** Structural Comparison of *Mononegavirales* Nucleocapsids.

	RABV	RSV	hMPV	PIV5	MeV	NiV	NDV	EBOV	MARV	BoDV
**VSV**	2.63/96 *	5.07/79	5.17/81	5.34/78	5.30/79	4.73/83	5.20/75	5.18/82	4.20/85	5.09/78
**RABV**		5.01/76	4.93/78	5.04/73	5.06/76	4.27/81	5.11/73	4.38/78	5.01/82	5.03/75
**RSV**			1.40/100	4.35/91	4.35/91	4.90/93	4.36/91	4.39/87	4.85/86	4.83/80
**hMPV**				4.10/94	4.20/94	4.79/92	4.24/94	4.48/89	4.11/90	4.36/78
**PIV5**					2.47/97	3.59/100	1.82/98	4.87/89	3.77/94	4.51/83
**MeV**						3.26/97	2.32/98	4.43/87	3.97/94	4.41/81
**NiV**							3.38/99	4.12/92	3.62/91	5.19/80
**NDV**								4.79/89	3.92/96	4.76/84
**EBOV**									1.87/99	5.53/83
**MARV**										5.47/84

* The root-mean-square deviation (RMSD, Å) is presented for each pair of the homologous structures that have a TM-score ≥0.5. The number under the bar is the percentage (%) of overlapping residues. Shaded cells indicate a high similarity (> 95% overlap). For pairs of structures that have a RMSD < 2.0 Å, one structure was determined using the other structure as the initial model.

**Table 3:** Structural Comparison of *Bunyavirales* Nucleocapsids.

	LACV	LEAV	SBV	TSWV	TOSV	SFTSV
**BUNV**	2.11/98	1.87/100	2.07/100	3.68/87	-	-
**LACV**		2.07/94	2.09/93	3.64/85	-	-
**LEAV**			1.66/99	3.96/89	-	-
**SBV**				3.52/88	-	-
**RVFV**					1.88/91	1.83/90
**TOSV**						2.65/94

**Table 4:** Structural Comparison of Other Nucleocapsids.

	HTNV	HAZV	KUPV	ERVEV	IFBV	IFDV	ISAV	PepMV
**ANDV**	1.94/95	-	-	-	-	-	-	-
**CCHFV**		1.77/100 ^†^	1.90/99	1.56/91	-	-	-	-
**HAZV**			1.56/97	1.32/89	-	-	-	-
**KUPV**				1.27/87	-	-	-	-
**IFAV**					2.29/95	3.21/94	3.49/84	-
**IFBV**						3.02/93	3.36/78	-
**IFDV**							3.58/76	-
**PapMV**								2.06/93

^†^ The nucleocapsid structure of HAZV, KUPV, and ERVEV was determined using that of CCHFV as the initial model. The head structure of these proteins is almost identical. The RMSD was calculated for the stalk region from these structures.

**Table 5:** Structural Comparison of Representative Nucleocapsid Lobes.

	VSV-N ^≠^	CCHFV-N	HTNV-C
**BUNV-N**	4.77/82	3.77/82	
**BUNV-C**			2.88/87
**PepMV-N**	3.28/61		

^≠^ The letter N or C after the virus code indicates the N- or C-terminal lobe.

## References

[1] Knipe D., Howley P., Fields B.N., Griffin D.E. (2001). Fields’ Virilogy: Principles of Virus Structure.

[2] Lefkowitz E.J., Dempsey D.M., Hendrickson R.C., Orton R.J., Siddell S.G., Smith D.B. (2018). Virus taxonomy: The database of the International Committee on Taxonomy of Viruses (ICTV). Nucleic Acids Res..

[3] Amarasinghe G.K., Ayllón M.A., Bào Y., Basler C.F., Bavari S., Blasdell K.R., Briese T., Brown P.A., Bukreyev A., Balkema-Buschmann A. (2019). Taxonomy of the order Mononegavirales: Update 2019. Arch. Virol..

[4] Amarasinghe G.K., Ayllón M.A., Bào Y., Basler C.F., Bavari S., Blasdell K.R., Briese T., Brown P.A., Bukreyev A., Balkema-Buschmann A. (2003). Crystal structure of the borna disease virus nucleoprotein. Structure.

[5] Dong S., Yang P., Li G., Liu B., Wang W., Liu X., Xia B., Yang C., Lou Z., Guo Y. (2015). Insight into the Ebola virus nucleocapsid assembly mechanism: Crystal structure of Ebola virus nucleoprotein core domain at 1.8 A resolution. Protein Cell.

[6] Liu B., Dong S., Li G., Wang W., Liu X., Wang Y., Yang C., Rao Z., Guo Y. (2017). Structural insight into nucleoprotein conformation change chaperoned by VP35 peptide in marburg virus. J. Virol..

[7] Song X., Shan H., Zhu Y., Hu S., Xue L., Chen Y., Ding W., Niu T., Gu J., Ouyang S. (2019). Self-capping of nucleoprotein filaments protects the Newcastle disease virus genome. eLife.

[8] Yabukarski F., Lawrence P., Tarbouriech N., Bourhis J.M., Delaforge E., Jensen M.R., Ruigrok R.W., Blackledge M., Volchkov V., Jamin M. (2014). Structure of Nipah virus unassembled nucleoprotein in complex with its viral chaperone. Nat. Struct. Mol. Biol..

[9] Gutsche I., Desfosses A., Effantin G., Ling W.L., Haupt M., Ruigrok R.W., Sachse C., Schoehn G. (2015). Structural virology: Near-atomic cryo-EM structure of the helical measles virus nucleocapsid. Science.

[10] Alayyoubi M., Leser G.P., Kors C.A., Lamb R.A. (2015). Structure of the paramyxovirus parainfluenza virus 5 nucleoprotein-RNA complex. Proc. Natl. Acad. Sci. USA.

[11] Renner M., Bertinelli M., Leyrat C., Paesen G.C., Saraiva de Oliveira L.F., Huiskonen J.T., Grimes J.M. (2016). Nucleocapsid assembly in pneumoviruses is regulated by conformational switching of the N protein. eLife.

[12] Tawar R.G., Duquerroy S., Vonrhein C., Varela P.F., Damier-Piolle L., Castagné N., MacLellan K., Bedouelle H., Bricogne G., Bhella D. (2009). Crystal structure of a nucleocapsid-like nucleoprotein-RNA complex of respiratory syncytial virus. Science.

[13] Albertini A.A., Wernimont A.K., Muziol T., Ravelli R.B., Clapier C.R., Schoehn G., Weissenhorn W., Ruigrok R.W. (2006). Crystal structure of the rabies virus nucleoprotein-RNA complex. Science.

[14] Green T.J., Zhang X., Wertz G.W., Luo M. (2006). Structure of the vesicular stomatitis virus nucleoprotein-RNA complex. Science.

[15] Hastie K.M., Liu T., Li S., King L.B., Ngo N., Zandonatti M.A., Woods V.L., de la Torre J.C., Saphire E.O. (2011). Crystal structure of the Lassa virus nucleoprotein-RNA complex reveals a gating mechanism for RNA binding. Proc. Natl. Acad. Sci. USA.

[16] Guo Y., Wang W., Sun Y., Ma C., Wang X., Wang X., Liu P., Shen S., Li B., Lin J. (2016). Crystal structure of the core region of hantavirus nucleocapsid protein reveals the mechanism for ribonucleoprotein complex formation. J. Virol..

[17] Arragain B., Reguera J., Desfosses A., Gutsche I., Schoehn G., Malet H. (2019). High resolution cryo-EM structure of the helical RNA-bound Hantaan virus nucleocapsid reveals its assembly mechanisms. eLife.

[18] Carter S.D., Surtees R., Walter C.T., Ariza A., Bergeron É., Nichol S.T., Hiscox J.A., Edwards T.A., Barr J.N. (2012). Structure, function, and evolution of the Crimean-Congo hemorrhagic fever virus nucleocapsid protein. J. Virol..

[19] Wang W., Liu X., Wang X., Dong H., Ma C., Wang J., Liu B., Mao Y., Wang Y., Li T. (2015). Structural and functional diversity of nairovirus-encoded nucleoproteins. J. Virol..

[20] Li B., Wang Q., Pan X., Fernández de Castro I., Sun Y., Guo Y., Tao X., Risco C., Sui S.F., Lou Z. (2013). Bunyamwera virus possesses a distinct nucleocapsid protein to facilitate genome encapsidation. Proc. Natl. Acad. Sci. USA.

[21] Reguera J., Malet H., Weber F., Cusack S. (2013). Structural basis for encapsidation of genomic RNA by La Crosse *Orthobunyavirus nucleoprotein*. Proc. Natl. Acad. Sci. USA.

[22] Niu F., Shaw N., Wang Y.E., Jiao L., Ding W., Li X., Zhu P., Upur H., Ouyang S., Cheng G. (2013). Structure of the *Leanyer orthobunyavirus* nucleoprotein-RNA complex reveals unique architecture for RNA encapsidation. Proc. Natl. Acad. Sci. USA.

[23] Dong H., Li P., Bottcher B., Elliott R.M., Dong C. (2013). Crystal structure of *Schmallenberg orthobunyavirus* nucleoprotein-RNA complex reveals a novel RNA sequestration mechanism. Rna.

[24] Raymond D.D., Piper M.E., Gerrard S.R., Skiniotis G., Smith J.L. (2012). *Phleboviruses encapsidate* their genomes by sequestering RNA bases. Proc. Natl. Acad. Sci. USA.

[25] Olal D., Dick A., Woods V.L., Jr Liu T., Li S., Devignot S., Weber F., Saphire E.O., Daumke O. (2014). Structural insights into RNA encapsidation and helical assembly of the Toscana virus nucleoprotein. Nucleic Acids Res..

[26] Jiao L., Ouyang S., Liang M., Niu F., Shaw N., Wu W., Ding W., Jin C., Peng Y., Zhu Y. (2013). Structure of severe fever with thrombocytopenia syndrome virus nucleocapsid protein in complex with suramin reveals therapeutic potential. J. Virol..

[27] Komoda K., Narita M., Yamashita K., Tanaka I., Yao M. (2017). Asymmetric trimeric ring structure of the nucleocapsid protein of tospovirus. J. Virol..

[28] Ye Q., Krug R.M., Tao Y.J. (2006). The mechanism by which influenza A virus nucleoprotein forms oligomers and binds RNA. Nature.

[29] Ng A.K., Lam M.K., Zhang H., Liu J., Au S.W., Chan P.K., Wang J., Shaw P.C. (2012). Structural basis for RNA binding and homo-oligomer formation by influenza B virus nucleoprotein. J. Virol..

[30] Donchet A., Oliva J., Labaronne A., Tengo L., Miloudi M., CA Gerard F., Mas C., Schoehn G., WH Ruigrok R., Ducatez M. (2019). The structure of the nucleoprotein of Influenza D shows that all Orthomyxoviridae nucleoproteins have a similar NPCORE, with or without a NPTAIL for nuclear transport. Sci. Rep..

[31] Zheng W., Olson J., Vakharia V., Tao Y.J. (2013). The crystal structure and RNA-binding of an orthomyxovirus nucleoprotein. PLoS Pathog..

[32] Yang S., Wang T., Bohon J., Gagné M.È., Bolduc M., Leclerc D., Li H. (2012). Crystal structure of the coat protein of the flexible filamentous papaya mosaic virus. J. Mol. Biol..

[33] Agirrezabala X., Méndez-López E., Lasso G., Sánchez-Pina M.A., Aranda M., Valle M. (2015). The near-atomic cryoEM structure of a flexible filamentous plant virus shows homology of its coat protein with nucleoproteins of animal viruses. eLife.

[34] Green T.J., Cox R., Tsao J., Rowse M., Qiu S., Luo M. (2014). Common mechanism for RNA encapsidation by negative-strand RNA viruses. J. Virol..

[35] Chapman M.S., Liljas L. (2003). Structural folds of viral proteins. Adv. Protein Chem..

[36] Pandit S.B., Skolnick J. (2008). Fr-TM-align: A new protein structural alignment method based on fragment alignments and the TM-score. BMC Bioinform..

[37] Luo M., Green T.J., Zhang X., Tsao J., Qiu S. (2007). Structural comparisons of the nucleoprotein from three negative strand RNA virus families. J. Virol..

[38] Wichgers Schreur P.J., Kormelink R., Kortekaas J. (2018). Genome packaging of the Bunyavirales. Curr. Opin. Virol..

[39] Sherman M.B., Freiberg A.N., Holbrook M.R., Watowich S.J. (2009). Single-particle cryo-electron microscopy of Rift Valley fever virus. Virology.

[40] DeLano W.L. (2015). PyMOL Molecular Graphic System.

[41] Desfosses A., Ribeiro E.A., Schoehn G., Blondel D., Guilligay D., Jamin M., Ruigrok R.W., Gutsche I. (2013). Self-organization of the vesicular stomatitis virus nucleocapsid into a bullet shape. Nat. Commun..

[42] Ge P., Tsao J., Schein S., Green T.J., Luo M., Zhou Z.H. (2010). Cryo-EM model of the bullet-shaped vesicular stomatitis virus. Science.

[43] Luo M., Green T.J., Zhang X., Tsao J., Qiu S. (2007). Conserved characteristics of the rhabdovirus nucleoprotein. Virus Res..

[44] Zhang X., Green T.J., Tsao J., Qiu S., Luo M. (2008). Role of intermolecular interactions of vesicular stomatitis virus nucleoprotein in RNA encapsidation. J. Virol..

[45] Arranz R., Coloma R., Chichón F.J., Conesa J.J., Carrascosa J.L., Valpuesta J.M., Ortín J., Martín-Benito J. (2012). The structure of native influenza virion ribonucleoproteins. Science.

[46] Moeller A., Kirchdoerfer R.N., Potter C.S., Carragher B., Wilson I.A. (2012). Organization of the influenza virus replication machinery. Science.

[47] Pflug A., Guilligay D., Reich S., Cusack S. (2014). Structure of influenza A polymerase bound to the viral RNA promoter. Nature.

[48] Leyrat C., Yabukarski F., Tarbouriech N., Ribeiro E.A., Jensen M.R., Blackledge M., Ruigrok R.W., Jamin M. (2011). Structure of the vesicular stomatitis virus N^0^-P complex. PLoS Pathog..

[49] Aggarwal M., Leser G.P., Kors C.A., Lamb R.A. (2018). Structure of the paramyxovirus parainfluenza virus 5 nucleoprotein in complex with an amino-terminal peptide of the phosphoprotein. J. Virol..

[50] Kirchdoerfer R.N., Abelson D.M., Li S., Wood M.R., Saphire E.O. (2015). Assembly of the Ebola Virus nucleoprotein from a chaperoned VP35 complex. Cell Rep..

[51] Landeras-Bueno S., Oda S.I., Norris M.J., Li Salie Z., Guenaga J., Wyatt R.T., Saphire E.O. (2019). Sudan Ebolavirus VP35-NP crystal structure reveals a potential target for pan-filovirus treatment. mBio.

[52] Raymond D.D., Piper M.E., Gerrard S.R., Smith J.L. (2010). Structure of the Rift Valley fever virus nucleocapsid protein reveals another architecture for RNA encapsidation. Proc. Natl. Acad. Sci. USA.

[53] Chenavas S., Estrozi L.F., Slama-Schwok A., Delmas B., Di Primo C., Baudin F., Li X., Crépin T., Ruigrok R.W. (2013). Monomeric nucleoprotein of influenza A virus. PLoS Pathog..

[54] Ding H., Green T.J., Lu S., Luo M. (2006). Crystal structure of the oligomerization domain of the phosphoprotein of vesicular stomatitis virus. J. Virol..

[55] Jenni S., Bloyet L.M., Diaz-Avalos R., Liang B., Whelan S., Grigorieff N., Harrison S.C. (2020). Structure of the vesicular stomatitis virus l protein in complex with its phosphoprotein cofactor. Cell Rep..

[56] Ruigrok R.W., Crepin T., Kolakofsky D. (2011). Nucleoproteins and nucleocapsids of negative-strand RNA viruses. Curr. Opin. Microbiol..

[57] Kolakofsky D., Roux L., Garcin D., Ruigrok R.W. (2005). Paramyxovirus mRNA editing, the “rule of six” and error catastrophe: A hypothesis. J. Gen. Virol..

[58] Kolakofsky D., Pelet T., Garcin D., Hausmann S., Curran J., Roux L. (1998). Paramyxovirus RNA synthesis and the requirement for hexamer genome length: The rule of six revisited. J. Virol..

[59] Iseni F., Baudin F., Garcin D., Marq J.B., Ruigrok R.W., Kolakofsky D. (2002). Chemical modification of nucleotide bases and mRNA editing depend on hexamer or nucleoprotein phase in Sendai virus nucleocapsids. Rna.

[60] Harouaka D., Wertz G.W. (2009). Mutations in the C-terminal loop of the nucleocapsid protein affect vesicular stomatitis virus RNA replication and transcription differentially. J. Virol..

[61] Harouaka D., Wertz G.W. (2012). Second-site mutations selected in transcriptional regulatory sequences compensate for engineered mutations in the vesicular stomatitis virus nucleocapsid protein. J. Virol..

[62] Anhlan D., Grundmann N., Makalowski W., Ludwig S., Scholtissek C. (2011). Origin of the 1918 pandemic H1N1 influenza A virus as studied by codon usage patterns and phylogenetic analysis. Rna.

[63] Cristina J., Moreno P., Moratorio G., Musto H. (2015). Genome-wide analysis of codon usage bias in Ebolavirus. Virus Res..

[64] Pagan I., Holmes E.C., Simon-Loriere E. (2012). Level of gene expression is a major determinant of protein evolution in the viral order Mononegavirales. J. Virol..

[65] Wang B., Yang C., Tekes G., Mueller S., Paul A., Whelan S.P., Wimmer E. (2015). Recoding of the vesicular stomatitis virus L gene by computer-aided design provides a live, attenuated vaccine candidate. mBio.

[66] Rima B.K. (2015). Nucleotide sequence conservation in paramyxoviruses; the concept of codon constellation. J. Gen. Virol..

[67] Gumpper R.H., Li W., Luo M. (2018). Contrains of viral RNA synthesis on codon usage of negative strand RNA virus. J. Virol..

[68] Gumpper R.H., Li W., Castañeda C.H., Scuderi M.J., Bashkin J.K., Luo M. (2018). A polyamide inhibits replication of vesicular stomatitis virus by targeting RNA in the nucleocapsid. J. Virol..

[69] Reich S., Guilligay D., Pflug A., Malet H., Berger I., Crépin T., Hart D., Lunardi T., Nanao M., Ruigrok R.W. (2014). Structural insight into cap-snatching and RNA synthesis by influenza polymerase. Nature.

[70] Hengrung N., El Omari K., Serna Martin I., Vreede F.T., Cusack S., Rambo R.P., Vonrhein C., Bricogne G., Stuart D.I., Grimes J.M. (2015). Crystal structure of the RNA-dependent RNA polymerase from influenza C virus. Nature.

[71] Fan H., Walker A.P., Carrique L., Keown J.R., Serna Martin I., Karia D., Sharps J., Hengrung N., Pardon E., Steyaert J. (2019). Structures of influenza A virus RNA polymerase offer insight into viral genome replication. Nature.

[72] Pflug A., Lukarska M., Resa-Infante P., Reich S., Cusack S. (2017). Structural insights into RNA synthesis by the influenza virus transcription-replication machine. Virus Res..

[73] Te Velthuis A.J., Fodor E. (2016). Influenza virus RNA polymerase: Insights into the mechanisms of viral RNA synthesis. Nat. Rev. Microbiol..

[74] Reguera J., Gerlach P., Cusack S. (2016). Towards a structural understanding of RNA synthesis by negative strand RNA viral polymerases. Curr. Opin. Struct. Biol..

[75] Gerlach P., Malet H., Cusack S., Reguera J. (2015). Structural Insights into bunyavirus replication and its regulation by the vRNA promoter. Cell.

[76] Biedenkopf N., Hartlieb B., Hoenen T., Becker S. (2013). Phosphorylation of Ebola virus VP30 influences the composition of the viral nucleocapsid complex: Impact on viral transcription and replication. J. Biol. Chem..

[77] Kirchdoerfer R.N., Moyer C.L., Abelson D.M., Saphire E.O. (2016). The Ebola Virus VP30-NP interaction is a regulator of viral RNA synthesis. PLoS Pathog..

[78] Green T.J., Luo M. (2009). Structure of the vesicular stomatitis virus nucleocapsid in complex with the nucleocapsid-binding domain of the small polymerase cofactor, P. Proc. Natl. Acad. Sci. USA.

[79] Kingston R.L., Baase W.A., Gay L.S. (2004). Characterization of nucleocapsid binding by the measles virus and mumps virus phosphoproteins. J. Virol..

[80] Bourhis J.M., Receveur-Bréchot V., Oglesbee M., Zhang X., Buccellato M., Darbon H., Canard B., Finet S., Longhi S. (2005). The intrinsically disordered C-terminal domain of the measles virus nucleoprotein interacts with the C-terminal domain of the phosphoprotein via two distinct sites and remains predominantly unfolded. Protein. Sci..

[81] Kingston R.L., Gay L.S., Baase W.S., Matthews B.W. (2008). Structure of the nucleocapsid-binding domain from the mumps virus polymerase; an example of protein folding induced by crystallization. J. Mol. Biol..

[82] Cox R., Pickar A., Qiu S., Tsao J., Rodenburg C., Dokland T., Elson A., He B., Luo M. (2014). Structural studies on the authentic mumps virus nucleocapsid showing uncoiling by the phosphoprotein. Proc. Natl. Acad. Sci. USA.

[83] Cox R., Green T.J., Purushotham S., Deivanayagam C., Bedwell G.J., Prevelige P.E., Luo M. (2013). Structural and functional characterization of the mumps virus phosphoprotein. J. Virol..

[84] Horwitz J.A., Jenni S., Harrison S.C., Whelan S.P.J. (2020). Structure of a rabies virus polymerase complex from electron cryo-microscopy. Proc. Natl. Acad. Sci. USA.

[85] Gilman M., Liu C., Fung A., Behera I., Jordan P., Rigaux P., Ysebaert N., Tcherniuk S., Sourimant J., Eléouët J.F. (2019). Structure of the respiratory syncytial virus polymerase complex. Cell.

[86] Pan J., Pan J., Qian X., Lattmann S., El Sahili A., Yeo T.H., Jia H., Cressey T., Ludeke B., Noton S. (2020). Structure of the human metapneumovirus polymerase phosphoprotein complex. Nature.

[87] Ogino M., Gupta N., Green T.J., Ogino T. (2019). A dual-functional priming-capping loop of rhabdoviral RNA polymerases directs terminal de novo initiation and capping intermediate formation. Nucleic Acids Res..

[88] Qanungo K.R., Shaji D., Mathur M., Banerjee A.K. (2004). Two RNA polymerase complexes from vesicular stomatitis virus-infected cells that carry out transcription and replication of genome RNA. Proc. Natl. Acad. Sci. USA.

[89] Ogino T., Green T.J. (2019). RNA Synthesis and capping by non-segmented negative strand RNA viral polymerases: Lessons from a prototypic virus. Front. Microbiol..

[90] Hodges J., Tang X., Landesman M.B., Ruedas J.B., Ghimire A., Gudheti M.V., Perrault J., Jorgensen E.M., Gerton J.M., Saffarian S. (2013). Asymmetric packaging of polymerases within vesicular stomatitis virus. Biochem. Biophys. Res. Commun..

[91] Liang B., Li Z., Jenni S., Rahmeh A.A., Morin B.M., Grant T., Grigorieff N., Harrison S.C., Whelan S. (2015). Structure of the L protein of vesicular stomatitis virus from electron cryomicroscopy. Cell.

[92] Guryanov S.G., Liljeroos L., Kasaragod P., Kajander T., Butcher S.J. (2016). Crystal structure of the measles virus nucleoprotein core in complex with an N-terminal region of phosphoprotein. J. Virol..

[93] Severin C., Terrell J.R., Zengel J.R., Cox R., Plemper R.K., He B., Luo M. (2016). Releasing the genomic RNA sequestered in the mumps virus nucleocapsid. J. Virol..

[94] Green T.J., Rowse M., Tsao J., Kang J., Ge P., Zhou H., Luo M. (2011). Access to RNA encapsidated in the nucleocapsid of vesicular stomatitis virus. J. Virol..

[95] Li W., Gumpper R.H., Uddin Y., Schmidt-Krey I., Luo M. (2018). Mutations in the nucleocapsid protein were complemented by mutations in the L protein to restore viral RNA synthesis. J. Virol..

[96] Abrescia N.G., Bamford D.H., Grimes J.M., Stuart D.I. (2012). Structure unifies the viral universe. Annu. Rev. Biochem..

[97] Rossmann M.G., Johnson J.E. (1989). Icosahedral RNA virus structure. Annu. Rev. Biochem..

